# Evaluation of Antibacterial, Antifungal, and Antioxidant Activities of Safflower Natural Dyes during Flowering

**DOI:** 10.1155/2014/762397

**Published:** 2014-06-22

**Authors:** Nidhal Salem, Kamel Msaada, Salem Elkahoui, Giuseppe Mangano, Sana Azaeiz, Imen Ben Slimen, Sarra Kefi, Giorgio Pintore, Ferid Limam, Brahim Marzouk

**Affiliations:** ^1^Laboratory of Bioactive Substances, Biotechnology Center in Borj-Cedria Technopol, BP 901, 2050 Hammam-Lif, Tunisia; ^2^Dipartimento di Scienze del Farmaco, Via Muroni, 2307100 Sassari, Italy

## Abstract

Two *Carthamus tinctorius* varieties (Jawhara and 104) were studied in order to investigate their natural dyes contents and biological activities. Obtained results showed that quinochalcone contents and antioxidant activities varied considerably as function of flowering stages. So flowers at fructification stage contained the highest carthamin content with the strongest antioxidant capacity with all assays (FRAP, DPPH, and chelating power methods). In parallel, we showed a decrease in the content of precarthamin. The quantitative variation of these molecules could be due to colour change of *C. tinctorius* flowers. Correlation analysis indicated that the ABTS method showed the highest correlation coefficients with carthamin and precarthamin contents, that is, 0.886 and 0.973, respectively. Concerning the regional effect, the contents of precarthamin and carthamin varied significantly (*P* < 0.05) at studied regions with the optimum production given by samples of Beja (902.41 *μ*g/g DW and 42.05 *μ*g/g DW, respectively, at flowering stage). During flowering, the antimicrobial activity of these two natural dyes increased where the maximum inhibitory effect mentioned with carthamin mainly against *E. coli* (iz = 25.89 mm) at fructification stage. Therefore, the increased frequency of resistance to commonly used antibiotics leads to the search for new effective natural drugs at food and pharmaceutical industries.

## 1. Introduction

Synthetic dye industry has trended to decline with the increasing awareness of toxicity and excessive use of artificial food additives. In fact, considerable interest has been emerged linking synthetic colorants intolerance with various environmental pollution and adverse toxicological side effects, particularly mental disorders. Therefore, several limitations and restrictions have been put in place for their use and their substitution by natural antioxidants [1]. Food additives are commonly used in processed food to improve appearance, flavor, taste, color, nutritive value, and conservation. The principal classes of these food additives are natural colorants [2]. In addition to their coloring properties, chalcones have generated intensive scientific interest due to their biological and industrial applications such as antibacterial, antifungal, insecticidal, anesthetic, anti-inflammatory, and analgesic effects [3]. These pigments are safe for food and have curative effects on diseases such as lack of oxygen, coronary heart diseases, myocardial infarction, and cerebral and renal thrombosis [4]. Hence, these dyes were reported to exert antioxidant and radical-scavenging activities and had been recently recommended for use as food colorants [5]. On the other hand, these natural colorants exhibited antibacterial and antifungal activities thanks to the existence of quinones in their structure contributing to the longer life of the products that are used in [6]. Nevertheless, production of these natural colorants in plant tissues is highly conducted by many extrinsic and intrinsic factors such as cultivar, variety, biotic and abiotic factors, ontogenetic stage, and growing region [7]. Many studies highlighted the correlation between the beneficial health qualities of these pigments and their high biological capacities since the change of these natural dyes may reflect their biological capacities during maturation [7, 8]. Therefore, controlled production of natural dyes appears to be a high priority and can be considered as a key factor towards their maximization and their high quality.

Among the sources of these natural dyes, safflower (*Carthamus tinctorius*. L), a member of Asteraceae family, is a famous traditional Chinese medicine which has many effects such as anticoagulant, vasodilator, antioxidant, immunosuppressant, and neuroprotector [9]. It is extensively used for producing food colorants. Safflower florets contain yellow and red quinochalcone natural dyes such as safflower yellow A, safflower yellow B, safflomin C, precarthamin, and carthamin [10]. These chalcones are the main constituents of glycosylated flavonoids in safflower which were not detected in other natural products. Carthamin, a red quinochalcone isolated from safflower, has been used extensively as a natural color additive for foods and cosmetics and as a nutraceutical in food industry [11]. Several studies were performed regarding environmental factors fluctuations and their consequences on the abundance of natural colorants, whereas there is unavailable research focusing on ontogenic and growing region effects on the biological activity and precarthamin and carthamin contents of safflower florets. Thus, in order to fully understand the nutritional value offered by safflower florets, the purpose of this work was (i) to purify, in order to quantify,* C. tinctorius* flower quinochalcone molecules, (ii) to ascertain the potential effects of safflower variety, growing region, and flowering stage on carthamin and precarthamin contents, and (iii) finally the antioxidant and antimicrobial activities of these purified molecules under the influence of these factors were evaluated. Simultaneously, the relationship between antioxidant capacity and the contents of these two natural colorants was discussed.

## 2. Materials and Methods

### 2.1. Chemicals and Reagents

Sephadex LH-20 was purchased from Amersham Bioscience. All solvents used in the experiments were purchased from LAB-SCAN. Chlorhydric acid (HCl), trifluoroacetic acid (TFA), butylated hydroxytoluene (BHT), ethylenediaminetetraacetic acid (EDTA), 3-(2-pyridyl)-5,6-bis(4-phenyl-sulphonic acid)-1,2,4-triazine (ferrozine), iron (II) chloride tetrahydrate (FeCl_2_ *·*4H_2_O), iron (II) chloride (FeCl_2_), iron (III) chloride (FeCl_3_), 1,1-diphenyl-2-picrylhydrazyl (DPPH), and TPTZ (2,4,6 tripyridyl-s-triazine) were purchased from Sigma-Aldrich (Steinheim, Germany).

### 2.2. Plant Material

Two varieties of* Carthamus tinctorius* flowers (Jawhara and 104) were harvested randomly from two different Tunisian localities: Beja (North Western Tunisia; latitude 36° 43′ 31.19′′ (N); 9° 11′ 14.52′′ E; altitude 225 m) and Tunis (latitude 36° 50′ 29.68′′ (N); longitude 10° 12′ 19.44′′ (E); 3 m elevation) at bud formation (Bu), flower formation (F), full flowering (FF), and seed formation (Se). The samples were freeze-dried and stored at −80°C until use. According to the isolated molecule, the flowers were separated into yellow and red flowers. The sampling was conducted six times and each sample was constituted with flowers with different colors.

### 2.3. Quinochalcone Extraction

#### 2.3.1. Isolation of Precarthamin

Yellow immature flowers of safflower collected from the two Tunisian regions and at different flowering stages were macerated with MeOH to remove yellow pigments. After filtration, the flowers were homogenized and extracted with 400 mL of acetone containing 1% (TFA). The filtrate was subjected to a Sephadex LH-20 column and eluted gradiently with 20–80% CH_3_CN/H_2_O containing 1% TFA. Precarthamin fractions were isolated by preparative reverse-phase HPLC and purified on an HPLC column (Column Develosil ODS-10/20; gradient elution: 30–50% CH_3_CN/H_2_O containing 1% TFA). The analysis of precarthamine fraction was lyophilized and analyzed by TLC (Merck RP C18, Rf 0.5, 60% MeOH/H_2_O) and HPLC (gradient 60% MeOH/H_2_O). The calibration curve was produced by the integration of absorption peaks generated from the analysis of dilution series of rutin trihydrate. The isolated precarthamin was identified by NMR spectrometry.

#### 2.3.2. Isolation of Carthamin

A fine powder of safflower flowers (collected from the two Tunisian regions and at different flowering stages) was solubilized in 20 mL of potassium carbonate (K_2_CO_3_) 0.5% (wv^−1^) under continuous stirring for 30 min at room temperature (21 ± 1°C). Different extracts obtained were acidified by citric acid to a concentration of 0.5% for the spectrophotometric analysis and purification. Hence, 0.5 g of cellulose was suspended in the solution previously obtained under continuous stirring. Successive centrifugations were made (15 min; 3500 rpm; 5°C) and the pellets were suspended in 10 mL of 60% acetone.

All acetonic extracts obtained were combined and concentrated to a small volume. The latter was passed through a column of Avicel cellulose (1.9 × 50 cm) with a mixture of n-butanol/acetic acid/water (4 + 2 + 1, vv^−1^). Carthamin was eluted with 60% acetone (vv^−1^) and then evaporated at 35°C to be purified again by a silica gel column (silica gel Davisil). The carthamin fraction was lyophilized and analyzed by spectrophotometry (310–600 nm), infrared (500–4000 cm^−1^), TLC (60% MeOH/H_2_O), and HPLC (60% MeOH/H_2_O) [12]. The calibration curve was produced by the integration of absorption peaks generated from the analysis of dilution series of rutin trihydrate.

### 2.4. Determination of Antioxidant Capacity

#### 2.4.1. DPPH Assay

Radical-scavenging activity of plant extracts against stable 2, 2 diphenyl 2 picrylhydrazyl hydrate (DPPH) was determined by the slightly modified method of Hatano et al. [13]. DPPH reacts with an antioxidant compound which can donate hydrogen and reduce DPPH. The change in colour (from deep violet to light yellow) was measured at 517 nm on a UV visible light spectrophotometer. The solution of DPPH in methanol 0.2 mM was prepared fresh daily before UV measurements. One-half milliliter of this solution was mixed with* C. tinctorius* purified quinochalcones (2 mL, 10–1000 *μ*g/mL). The samples were kept in the dark for 15 minutes at room temperature and the decrease in absorbance was measured. The experiment was carried out in triplicate. Radical-scavenging activity was calculated by the following formula:

(1) $$\begin{array}{c} \text{IP}\%=\left[\frac{\left(A_{\text{blank}}-A_{\text{sample}}\right)}{A_{\text{blank}}}\right]\times 100, \end{array}$$

where *A*_blank_ is the absorbance of the control reaction and *A*_sample_ is the absorbance in the presence of purified molecules. Extract concentration providing 50% inhibition (IC_50_) was calculated from the regression equation prepared from the concentration of the extracts and the inhibition percentage. BHT was used as a positive control.

#### 2.4.2. Metal-Chelating Power

According to Zhao et al. (2006) [14], 0.1 mL of flower extracts was added to 0.05 mL of 2 mM FeCl_2_. The reaction was initiated by the addition of 0.1 mL 5 mM ferrozine and 2.75 mL of distilled water. The mixture was shaken vigorously and left at room temperature for 10 min. The absorbance of the solution was then measured at 562 nm. The scavenging activity was calculated as follows:

(2) $$\begin{array}{c} \text{IP}\%=\left[\frac{\left(A_{\text{blank}}-A_{\text{sample}}\right)}{A_{\text{blank}}}\right]\times 100, \end{array}$$

where *A*_blank_ is the absorbance of the control reaction and *A*_sample_ is the absorbance in the presence of plant extract. IC_50_ was calculated from the plot of inhibition percentage against extract concentration. EDTA was used as a positive control.

#### 2.4.3. Ferric Reducing Antioxidant Power (FRAP) Assay

The FRAP assay was done according to Benzie and Strain [15] with some modifications. The stock solutions included 300 mM acetate buffer (3.1 g CH_3_COONa*·*3H_2_O and 16 mL CH_3_COOH), pH 3.6, 10 mM hydrochloric acid, and 20 mM ferric chloride hexahydrate solution. The fresh working solution was prepared by mixing 25 mL acetate buffer, 2.5 mL TPTZ solution, and 2.5 mL FeCl_3_ *·*6H_2_O solution and then warmed at 37°C before using. The solution of* C. tinctorius* samples 500 *μ*g/mL and that of trolox were formed in methanol. 10 *μ*L of each of the samples solutions was taken in separate test tubes and 2990 *μ*L of FRAP solution was added in each to make total volume up to 3 mL. The quinochalcones samples were allowed to react with FRAP solution in the dark for 30 minutes. Reading of the coloured product (ferrous tripyridyltriazine complex) was then taken at 593 nm by UV visible spectrophotometer. The FRAP values were determined as micromoles of trolox equivalents per mL of sample by computing with standard calibration curve constructed for different concentrations of trolox. Results were expressed in TE *μ*M/mL.

### 2.5. Screening of Antibacterial and Antifungal Activities

Antibacterial activity was analyzed by the disc diffusion method [16] against five human pathogenic bacteria including* Bacillus cereus *ATCC 14759, methicillin-resistant* Staphylococcus aureus*,* E. coli *ATCC 25218,* Staphylococcus aureus *ATCC 25923, and* Pseudomonas aeruginosa *ATCC 27853. All bacteria were grown on Mueller-Hinton plate at 30°C for 18–24 h of previous inoculation onto the nutrient agar. A loop of bacteria from the agar slant stock was cultivated in nutrient broth overnight and spread with a sterile cotton swap onto Petri dishes containing 10 mL of API suspension medium and adjusted to the 0.5 McFarland turbidity standards with a Densimat (BioMerieux). Sterile filter paper discs (6 mm in diameter) impregnated with plant extract were placed on the cultured plates. After 1-2 h at 4°C, the treated Petri dishes were incubated at 25 or 37°C for 18–24 h. Gentamicin was used as the positive one. The antimicrobial activity was evaluated by measuring the diameter of the growth inhibition zone around the discs. Each experiment was carried out in triplicate and the mean diameter of the inhibition zone was recorded. The same agar-disc diffusion method was used for screening the antifungal activity of* C. tinctorius* carthamin and precarthamin. One yeast strain (*Candida albicans*) was first grown on Sabouraud chloramphenicol agar plate at 30°C for 18–24 h. Several colonies of similar morphology of the clinical yeast were transferred into Api suspension medium and adjusted to 2 McFarland turbidity standard with a Densimat (Bio-Merieux). The inocula of the respective yeast were streaked onto Sabouraud chloramphenicol agar plates at 30°C using a sterile swab and then dried. The treated Petri dishes were placed at 4°C for 1-2 h and then incubated at 37°C for 18–24 h. The inhibition of fungal growth was also evaluated by measuring the diameter of the transparent inhibition zone around each disc. The average of three measurements was taken. The susceptibility of the standard was determined using a disc paper containing 300 *μ*g of nystatin.

### 2.6. Statistical Analysis

All analyses were performed in triplicate, and the results are expressed as mean values (standard deviations (SD)). The data were subjected to statistical analysis using statistical program package STATISTICA [17]. The one-way analysis of variance (ANOVA) followed by the Duncan multiple range test was employed and the differences between individual means and each solvent used were deemed to be significant at *P* < 0.05.

## 3. Results and Discussion

### 3.1. Varietal Effect on Quinochalcones Production

The purification of chalcones belonging to glycosylated flavonoids yielded two natural pigments which are characteristics of safflower: the precarthamine (C_44_H_43_O_24_) and carthamin (C_43_H_42_O_22_). The contents of these bioactive colorants and their antioxidant capacities may be significantly (*P* < 0.05) different among these two safflower varieties (“104” and “Jawhara”). Some differences existed in carthamin and precarthamin contents of* C. tinctorius* flower at flowering stage, where the highest contents were found in “Jawhara” with reporting values of 902.41 ± 0.28 *μ*g/g DW (precarthamine) and 42.05 ± 1.52 *μ*g/g DW (carthamin). The lowest contents were detected in “104” with values of 866.11 ± 0.58 *μ*g/g DW (precarthamin) and 35.22 ± 0.23 *μ*g/g DW (carthamin) (Table 1). This difference could be determined by genetic factors and environmental conditions.

All varieties showed the highest radical-scavenging activity compared to the BHT standard. In fact, “Jawhara” had the highest scavenging activity with the IC_50_ value of 1.23 ± 0.12 *μ*g/mL (carthamin) and 2.98 ± 0.54 *μ*g/mL (precarthamin) while 104 had the lowest one (IC_50_ did not exceed 3.15 ± 0.04 *μ*g/mL). Concerning chelating ability assay, these two purified quinochalcones appeared to be better chelators of ferrous irons than the positive control EDTA (IC_50_ = 100.00 ± 0.01) with IC_50_ ranging from 9.23 ± 0.29 to 12.33 ± 0.25 *μ*g/mL. In addition, there was found a significant (*P* < 0.05) difference in FRAP between the two studied varieties and “Jawhara” exhibited the highest value of 210.33 ± 0.25 *μ*M/mL TE. Therefore, our data showed that the variation of antioxidant activity was variety-dependent.

The lowest antioxidant activity observed in the “104” variety was perhaps attributed to the lower quinochalcones contents by comparison to “Jawhara” (Table 1), since quinochalcone C-glycosides were known for their strong antioxidant activity [18, 19]. Nevertheless, there is no publication focusing on the content change of these purified quinochalcones among safflower varieties.

Independently of safflower variety, this strong activity of carthamin and precarthamin isolated from* C. tinctorius* flowers was found to be due to the presence of reactive *α*- and *β*-unsaturated keto group in the chalcone structure. Moreover, as metal chelators, these flavonoids play an important role in both the bioavailability and the toxicity of a variety of metals [20].

### 3.2. Regional Effect on Quinochalcones Production

Although several studies have been focused on the identification of floral pigments of safflower [21], no study has focused on determining the regional effect on their composition. The bioactive components contents and their antioxidant capacity in two different regions in Tunisia varied greatly (Table 2).

At flowering stage, the contents of precarthamin varied significantly (*P* < 0.05) at studied regions with the optimum production given by samples of Beja (902.41 *μ*g/g DW) compared to 789.86 *μ*g/g DW in the region of Tunis.

The precarthamin amount was higher than those of other flavonoids isolated from flowers of* C. tinctorius* such as quercetin 3-galactoside (10.53 *μ*g/100 g MS) and gallic acid (88.41 *μ*g/100 g MS) [7].

As for precarthamin, the carthamin content varied significantly (*P* < 0.05) depending on the region with 42.05 *μ*g/g DW and 25.97 *μ*g/g DW at the regions of Beja and Tunis, respectively (Table 2). Hence, these quantitative variations could be due to the changes in the environmental factors such as temperature, rainfall, and humidity of the two regions [22]. Both natural pigments (carthamin and precarthamin) are known for their antiulcer, antihistamine, anti-inflammatory, antimicrobial, and cytotoxic capacities. All these effects and others derive from their powerful antioxidant activity [23]. In this context, the results of Table 2 illustrated a significant (*P* < 0.05) variation in the antioxidant activity between samples of* C. tinctorius* from studied locations. The samples collected from Beja showed the highest antiradical activity (1.23 *μ*g/mL and 2.98 *μ*g/mL for carthamin and precarthamin, resp.) and the main chelating power (9.23 *μ*g/mL and 11.01 *μ*g/mL for carthamin and precarthamin, resp.). In agreement with DPPH and chelating power, we can consider that samples collected from Beja possessed much higher FRAP than those collected fromTunis for the two purified molecules.

These findings were in agreement with Farhat et al. [24] results, which showed that samples cultivated in the coastal regions Kelibia and Soliman had higher antiradical activity than Bou Arada and Sers. However, differences between samples collected from different localities were not significant for FRAP test. Hence, variation in environmental factors affected the composition of these natural pigments and consequently their antioxidative ability.

### 3.3. Relationship between Quinochalcone Contents and Antioxidant Activity

The relationship between carthamin and precarthamin contents and antioxidant activity of safflower flowers was shown in Table 3. The results indicated that there was a positive and highly significant (*P* < 0.05) relationship between quinochalcones content and antioxidant activity with different assays, especially among carthamin and precarthamin contents and antioxidant capacities based on FRAP test (*r* ^2^ = 0.98). The lowest correlation was found between carthamin content and antioxidant activity obtained by chelating power (*r* ^2^ = −0.96). The antioxidant activity of these two natural dyes is mainly due to their redox properties, which allow them to act as chelating agents, hydrogen donors, and singlet oxygen quenchers [25]. The results may improve the nutritional value of these colorants as additives in the food industry.

### 3.4. The Effect of Developmental Stages on Carthamin, Precarthamin, and Antioxidant Activity

**Figure 1:**
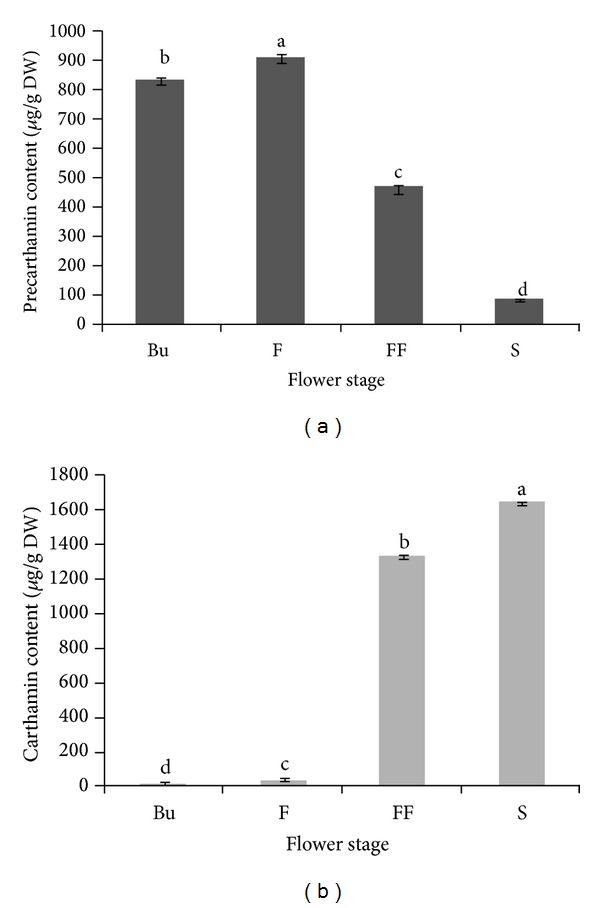
Concentrations of precarthamin (a) and carthamin (b) (*μ*g/g DW) at four flowering stages. Different letters (a–d) denote statistically significant differences by Duncan's multiple range test at *P* < 0.05.

The composition of bioactive purified quinochalcones and their antioxidant capacity in safflower were significantly (*P* < 0.05) affected by the developmental stages. In fact, flower at fructification stage contained the highest carthamin content (1641.23 ± 0.89 DW) (Figure 1) and showed the strongest antioxidant capacity with different assays (Table 4). Meanwhile, in parallel, there is a decrease in the content of precarthamin which was more pronounced at the fructification stage (110th DARE) with a value of 83.37 ± 1.78 with the lowest antioxidant capacity.

The quantitative variation of these two molecules could be due to colour change of* C. tinctorius* flowers. In fact, the latter showed yellow colour during the flowering stage and gradually changed to red. The color transition of safflower is due to the conversion of yellow pigments (precarthamin) to a red pigment (carthamin) [10].

To the best to our knowledge, there is no information available on safflower quinochalcones during flower development. Salem et al. [7] found that other individual flavonoids in the same plant (*C. tinctorius*) such as rutin trihydrate followed the same profile with a gradual increase in their contents and antioxidant activity during flower development. Hence, several factors might explain this gradation such as cell division, cellular differentiation, shifts in membrane permeability, cell elongation, and a wide range of gene expression in association with changes in concentration of endogenous plant growth regulators and secondary metabolites [26]. Further, the results were in good agreement with the findings of Mahmood et al. [27] who found that the concentrations of myricetin and kaempferol also increased during strawberry ripening. However, rutin and quercetin contents gradually declined with maturity stage with* Lycopersicon esculentum* [28].

On the other hand, the higher antioxidant activity (antiradical activity, FRAP, and chelating power) could be explained by their higher content of carthamin especially at fructification stage (Table 4). This red pigment of safflower is enzymatically synthesized from a yellow precursor, precarthamin, at the late-blooming stage and accumulates in mature petals [10]. Therefore, it is suggested that the best harvesting time for* C. tinctorius* flowers may be at stages 1 and 4.

### 3.5. The Effect of Developmental Stages on Antimicrobial Activity of Carthamin and Precarthamin

Antimicrobial activity of the two quinochalcones, precarthamin and carthamin, from different stages of maturity was tested against three Gram-positive bacteria (*Escherichia coli*,* Staphylococcus aureus*, and* Bacillus cereus*), one Gram-negative bacterium (*Pseudomonas aeruginosa*), and one yeast strain (*Candida albicans*).

As shown in Table 5, the carthamin exhibited relatively strong antibacterial activity during flower stages against various bacterial strains studied where the inhibition zone (iz) reached up to 26 mm mainly against* E. coli*, a value which was similar to gentamicin (iz = 26). In contrast, the precarthamin exhibited the least antibacterial activity against the selected bacterial strains where the iz did not exceed 17.56 mm (Table 6). Hence, the activity of these two chalcone compounds can be related to molecular hydrophobicity and charges on C atom at position 3 (C3) explaining the antimicrobial activity differences [11].

However, these two molecules exerted a moderate (carthamin) or ineffective (precarthamin) antibacterial activity against* Staphylococcus aureus *(Tables 5 and 6). This may be due to the presence of hydroxyl groups at positions 2 and 3 in the ring B of chalcone molecule characterizing these two natural pigments of safflower [29]. However, Patel and Rao [30] reasoned that the lack of activity can be proven by using large doses.

Concerning the antifungal activity of these two pigments, carthamin (iz varying from 9.20 to 12.03 nm) was more active against* Candida albicans* than precarthamin (iz varying from 7.11 to 10.20 nm). The antifungal action of these chalcones has been largely attributed to the reactive enone moiety [31]. However, the same authors previously reported that the potency of the chalcones against* C. albicans* to a large extent depended on their ability to interact with sulphydryl groups. So further studies are needed to ascertain safflower chalcone action.

During flowering, the antimicrobial activity of these two quinochalcones gradually increased where the fructification stage showed the maximum inhibitory activity with carthamin (Table 5). These data showed a correlation between the concentration of carthamin and its antimicrobial activity. Nevertheless, no correlation among precarthamin concentrations and antimicrobial activity was observed during flower development (Table 6). In fact, the maximum activity of this molecule was viewed at fructification stage (antibacterial activity) and full flowering stage (antifungal activity).

The presence of *α*- and *β*-unsaturated carbonyl systems makes these chalcones more active and facilitates their use in the food industry [32]. On the other hand, safflower is susceptible to several fungal diseases such as alternaria, wilt, and rust. Thus, the use of plant extracts with antifungal power is a promising alternative against these diseases [33]. Hence, further experiments were performed to investigate the antimicrobial action of precarthamin and carthamin.

## 4. Conclusion

In conclusion, our findings revealed that* C. tinctorius* flowers at fructification stage showed the highest carthamin content with the strongest antioxidant and significant antimicrobial activities, suggesting that this stage was the best harvesting time of safflower. Moreover, this study can be considered as the first report focusing on biological activities of safflower natural quinochalcones as influenced by environmental factors. These findings underlined the potential consumption of safflower as a suitable source of natural dyes as an alternative to food synthetic colorants.

## Figures and Tables

**Table 1:** Precarthamin and carthamin contents (*μ*g/g DW) and antioxidant capacities in flowers of two *C. tinctorius* varieties at flowering stage.

Isolated molecule	Content (*μ*g/g DW)	Variety	Antioxidant activity		
			FRAP^A^	DPPH^B^	Chelating power^C^
Carthamin	42.05 ± 1.52^a^	“Jawhara”	210.33 ± 0.25^a^	1.23 ± 0.12^b^	9.23 ± 0.29^b^
	35.22 ± 0.23^b^	“104”	115.15 ± 0.02^b^	2.33 ± 0.52^a^	10.33 ± 0.01^a^
Precarthamin	902.41 ± 0.28^a^	“Jawhara”	98.26 ± 0.01^a^	2.98 ± 0.54^b^	11.01 ± 0.51^b^
	866.11 ± 0.58^b^	“104”	83.27 ± 0.74^b^	3.15 ± 0.04^a^	12.33 ± 0.25^a^

Synthetic antioxidants					

BHT				16.00 ± 0.02	
EDTA					100.00 ± 0.01
Blank			52.11 ± 0.01		

^A^The FRAP values were expressed in TE *μ*M/mL and ^B,C^IC_50_ values were expressed in *μ*g/mL. Data are reported as means ± SD (*n* = 3) and compared to control (C). ANOVA is followed by Duncan multiple range test (*P* < 0.05). Values in the same column with different superscripts (a-b) are significantly different at *P* < 0.05.

**Table 2:** Precarthamin and carthamin contents (*μ*g/g DW) and antioxidant capacities in flowers of *C. tinctorius* at two Tunisian regions at flowering stage.

Compound	Content (*µ*g/g DW)	Region	Antioxidant activity		
			FRAP^A^	DPPH^B^	Chelating power^C^
Carthamin	42.05 ± 1.52^a^	Beja	210.33 ± 0.25^a^	1.23 ± 0.12^b^	9.23 ± 0.20^b^
	25.97 ± 0.72^b^	Tunis	166.01 ± 0.55^b^	1.86 ± 0.55^a^	10.88 ± 0.06^a^
Precarthamin	902.41 ± 1.52^a^	Beja	98.26 ± 0.01^a^	2.98 ± 0.54^b^	11.01 ± 0.29^b^
	789.86 ± 0.78^b^	Tunis	72.14 ± 0.58^b^	4.01 ± 0.01^a^	15.89 ± 0.28^a^

Synthetic antioxidants					

BHT				16.00 ± 0.02	
EDTA					100.00 ± 0.01
Blank			52.11 ± 0.01		

^A^The FRAP values were expressed in TE *μ*M/mL and ^B,C^IC_50_ values were expressed in *μ*g/mL. Data are reported as means ± SD (*n* = 3) and compared to control (C). ANOVA is followed by Duncan multiple range test (*P* < 0.05). Values in the same column with different superscripts (a-b) are significantly different at *P* < 0.05.

**Table 3:** Correlation analysis between carthamin, precarthamin, and antioxidant capacity.

Antioxidant capacity	Carthamin	Precarthamin
FRAP	*r* ^2^ = 0.98	*r* ^2^ = 0.98
DPPH	*r* ^2^ = −0.98*	*r* ^2^ = −0.98
Chelating power	*r* ^2^ = −0.96*	*r* ^2^ = −0.98

*P < 0.05.

**Table 4:** Antioxidant capacity of natural quinochalcones of *C. tinctorius* flowers during flowering.

Flowering stage	Antioxidant activity					
	FRAP^A^		DPPH^B^		Chelating power^C^	
	Precar	Carth	Precar	Carth	Precar	Carth
Bu	103.23 ± 0.47^a^	102.23 ± 0.08^d^	2.25 ± 1.02^c^	1.56 ± 0.17^a^	11.09 ± 0.04^c^	10.99 ± 0.22^a^
F	98.26 ± 0.01^b^	210.33 ± 0.25^c^	2.98 ± 0.25^b^	1.23 ± 0.19^b^	11.01 ± 0.03^c^	9.23 ± 0.17^c^
FF	86.27 ± 0.10^c^	500.29 ± 0.11^b^	3.25 ± 0.58^a^	1.02 ± 0.44^c^	11.87 ± 0.07^b^	9.55 ± 0.89^b^
Se	56.22 ± 0.03^d^	589.27 ± 0.19^a^	3.58 ± 0.28^a^	0.86 ± 0.11^d^	12.02 ± 0.10^a^	8.02 ± 0.27^d^

Synthetic antioxidants						

BHT			16.00 ± 0.02			
EDTA					100.00 ± 0.01	
Blank	52.11 ± 0.01					

Precar: precarthamin; Carth: carthamin. Bu: bud formation, F: flower formation, FF: full flowering, and Se: seed formation. ^A^The FRAP values were expressed in TE *μ*M/mL and ^B,C^IC_50_ values were expressed in *μ*g/mL. Data are reported as means ± SD (*n* = 3) and compared to control (C). ANOVA is followed by Duncan multiple range test (*P* < 0.05). Values in the same column with different superscripts (a–d) are significantly different at *P* < 0.05.

**Table 5:** Antibacterial and antifungal capacities of carthamin isolated from *C. tinctorius* flowers during flowering.

Flowering stages	Bacterial strains										Yeast strains	
	*Ba* *ci* *ll* *us* *cereus* ATCC 14759		*methicillin-resistant Staphylococcus aureus *		*E. coli* ATCC 25218		*Staphylococcus aureus* ATCC 25923		*Pseudomonas aeruginosa* ATCC 27853		*Ca* *nd* *id* *a* *albicans*	
	IZ	MIC	IZ	MIC	IZ	MIC	IZ	MIC	IZ	MIC	IZ	MIC
Bu	15.02 ± 0.11^d^	151 ± 0.92^d^	8.23 ± 0.47^d^	199 ± 0.27^d^	22.44 ± 0.22^c^	143 ± 0.36^d^	nd	nd	14.02 ± 0.23^b^	163 ± 0.58^d^	9.20 ± 0.05^c^	229 ± 0.71^d^
F	19.01 ± 0.09^c^	145 ± 0.84^d^	12.03 ± 0.55^c^	155 ± 0.12^d^	24.88 ± 0.10^b^	140 ± 0.77^d^	6 ± 0.22^b^	210 ± 0.64^d^	13.22 ± 0.56^c^	166 ± 0.88^d^	10.22 ± 0.36^b^	227 ± 0.86^d^
FF	20.05 ± 0.04^b^	142 ± 0.88^d^	16.52 ± 0.08^b^	149 ± 0.22^d^	25.50 ± 0.04^a^	140 ± 0.53^d^	7.04 ± 0.55^a^	215 ± 0.13^d^	14.98 ± 0.47^b^	162 ± 0.84^d^	10.89 ± 0.55^b^	225 ± 0.19^d^
Se	22.03 ± 0.55^a^	144 ± 0.70^d^	18.56 ± 0.58^a^	147 ± 0.55^d^	25.89 ± 0.00^a^	138 ± 0.99^d^	7.69 ± 0.03^a^	215 ± 0.55^d^	15.23 ± 0.89^a^	160 ± 0.55^d^	12.03 ± 0.66^a^	222 ± 0.33^d^

Synthetic standards												

Gentamicin	22		25		28		35		23		—	
Nystatin	—		—		—		—		—		25	

Bu: bud formation, F: flower formation, FF: full flowering, and Se: seed formation. nd: not detected. Data are reported as means ± SD (*n* = 3) and compared to control (C). ANOVA is followed by Duncan multiple range test (*P* < 0.05). Values in the same column with different superscripts (a–d) are significantly different at *P* < 0.05.

**Table 6:** Antibacterial and antifungal capacities of precarthamin isolated from *C. tinctorius* flowers during flowering.

Flowering stage	Bacterial strains										Yeast strains	
	*Ba* *ci* *ll* *us* *cereus* ATCC 14759		*methicillin-resistant Staphylococcus aureus *		*E. coli* ATCC 25218		*Staphylococcus aureus* ATCC 25923		*Pseudomonas aeruginosa* ATCC 27853		*Ca* *nd* *id* *a* *albicans*	
	IZ	MIC	IZ	MIC	IZ	MIC	IZ	MIC	IZ	MIC	IZ	MIC
Bu	11.26 ± 0.55^a^	168 ± 0.23^c^	nd	nd	13.00 ± 0.55^c^	156 ± 0.28^b^	nd	nd	12.00 ± 0.89^c^	159.5 ± 0.96^c^	7.11 ± 0.37^c^	240 ± 0.24^a^
F	8.58 ± 0.74^d^	201 ± 0.54^a^	10.08 ± 0.55^c^	166 ± 0.74^a^	13.50 ± 0.69^c^	159 ± 0.11^a^	nd	nd	10.39 ± 0.22^d^	167 ± 0.55^a^	8.99 ± 0.07^b^	235 ± 0.33^b^
FF	9.89 ± 0.03^c^	199 ± 0.11^b^	12.30 ± 0.56^b^	160 ± 0.0^b^	15.23 ± 0.57^b^	152 ± 0.34^c^	nd	nd	13.37 ± 0.83^b^	160.25 ± 0.63	10.20 ± 0.78^a^	230 ± 0.14^d^
Se	10.05 ± 0.88^b^	165 ± 0.14^d^	13.20 ± 0.23^a^	158 ± 0.20^b^	16.89 ± 1.31^a^	150 ± 0.18^d^	nd	nd	15.93 ± 0.27^a^	153 ± 0.74^d^	9.03 ± 0.23^b^	233 ± 0.22^c^

Synthetic standards												

Gentamicin	22	—	25	—	28	—	35	—	23	—	—	—
Nystatin	—	—	—	—	—	—	—	—	—	—	25	—

Bu: bud formation, F: flower formation, FF: full flowering, and Se: seed formation. nd: not detected. Data are reported as means ± SD (*n* = 3) and compared to control (C). ANOVA is followed by Duncan multiple range test (*P* < 0.05). Values in the same column with different superscripts (a–d) are significantly different at *P* < 0.05.
